# The Ischemic Immature Brain: Views on Current Experimental Models

**DOI:** 10.3389/fncel.2018.00277

**Published:** 2018-08-29

**Authors:** Tânia Faustino-Mendes, Marta Machado-Pereira, Miguel Castelo-Branco, Raquel Ferreira

**Affiliations:** ^1^Faculty of Health Sciences, University of Beira Interior, Covilhã, Portugal; ^2^Health Sciences Research Centre (CICS-UBI), University of Beira Interior, Covilhã, Portugal; ^3^Hospital Center of Cova da Beira, Covilhã, Portugal

**Keywords:** perinatal, neonatal, stroke, experimental models, immature brain

## Stroke in preterm and term newborns

Perinatal stroke occurs between the 20th week of gestation and the 28th day after birth (Nelson, 2007). Brain injury within this period can also lead to conditions such as neonatal encephalopathy or to encephalopathy of prematurity. Considering the complex diagnosis and limited data available, the incidence of 1/2,300 live births is a likely underestimation (Nelson and Lynch, 2004; Lee et al., 2005). Nevertheless, these numbers are comparable to those in the elderly (Fernández-López et al., 2014). The most common subtypes are arterial ischemic stroke (Fernández-López et al., 2014), which induces a focal lesion similar to adult stroke, and cerebral sinovenous thrombosis (Govaert et al., 2009). Focal interruption of arterial or venous cerebral blood flow is usually secondary to thrombosis or embolism, with a multifactorial pathophysiology. Importantly, in the fetal circulatory system, placental or systemic venous emboli may pass through a patent *ductus arteriosus* or *foramen ovale* directly to the left carotid artery and subsequently to the left MCA, facilitating occlusion (Gunny and Lin, 2012). Confirmation by imaging or neuropathological studies is always mandatory (Govaert et al., 2009). Different risk factors have been recognized for perinatal stroke (Supplementary Table 1; Kurnik et al., 2003; Mirabelli-Badenier et al., 2012; Kasdorf and Perlman, 2013; Basu, 2014; Fernández-López et al., 2014; Kratzer et al., 2014; Machado et al., 2015; Buerki et al., 2016) but inflammation seems to be a prevalent underlying mechanism (Vexler and Yenari, 2009; Hagberg et al., 2015). For instance, chorioamnionitis, a bacterial infection of the amniochorionic membranes (Kasdorf and Perlman, 2013; Buerki et al., 2016) often leads to a longer labor period and worse prognosis (Vexler and Yenari, 2009). Nevertheless, although there is a substantial number of studies concerning neonatal encephalopathy (or hypoxic-ischemia encephalopathy), human data on perinatal stroke can be disparate; some authors found a positive correlation with pro-inflammatory polymorphisms, others did not (Hagberg et al., 2015).

Adult and perinatal stroke also cause distinct presenting symptoms: adults tend to present unilateral symptoms and only 3% have seizures; seizures are one of the most common presentations after perinatal stroke (Fernández-López et al., 2014). Hemiplegic cerebral palsy is also the most frequent long-term motor outcome of the latter (Nelson, 2007). However, several aspects delay the suspicion of diagnosis, since (i) newborns with seizures may appear clinically well-between episodes; (ii) initially, newborns may present discrete non-specific symptoms like lethargy, apnea, difficult feeding and impaired chewing; (iii) and some cases may be asymptomatic, presenting lateralized symptoms only around the 5th month. In fact, lateralized symptoms are rare in neonates (Nelson, 2007; Fernández-López et al., 2014). In 2011, Harbert and colleagues conducted the first human study demonstrating the positive effect of therapeutic hypothermia on perinatal stroke: active whole-body cooling *via* a blanket cooling device led to a significantly lower frequency of seizures (Harbert et al., 2011). Since these symptoms are associated to a worse prognosis, the risk of long-term neurologic disability is likely reduced. Given the complex pathophysiology and difficulty in obtaining an early and accurate diagnosis, new therapies are being tested, alone or in combination with hypothermia, to improve global outcome. Some include administration of growth factors, anticoagulant and antiplatelet agents, blood uric acid lowering medication, antioxidant and anti-inflammatory molecules, stem cells-based therapy and electrical stimulation (Cnossen et al., 2009; Gonzalez and Ferriero, 2009; Mirabelli-Badenier et al., 2012; Basu, 2014; Fernández-López et al., 2014; Kratzer et al., 2014).

## *In vitro* approaches

*In vitro* models resort to oxygen and glucose deprivation (OGD), followed by reoxygenation and nutrient replenishment. Since oxygen levels should be kept preferably under 2%, to represent the ischemic core, and around 7% if studying the ischemic penumbra (Tornabene and Brodin, 2016), these models can be very relatable while allowing easy assessment of cell activity, protein expression and release, and barrier properties of particular cell type(s). We have exposed either individualized cells or brain tissue, namely organotypic brain slice cultures (OSC), to very low O_2_ rates (0.1%; Ferreira et al., 2016; Machado-Pereira et al., 2017). These tissue cultures provide unique characteristics and several advantages over cell models, since they preserve whole organ structure and maintain neuronal activity and synapse circuitry. OSC also maintain integrity *in vitro* for over 2 weeks allowing a series of pharmacological studies. Other advantages include the possibility of using younger animals if needed (e.g., 1–3-day-old mice), the refinement of experimental doses/conditions and the reduction of the number of animals for *in vivo* models. To the best of our knowledge only one group has used OSC, from P8-10 rats (Leonardo et al., 2009). A considerable drawback from OSC, and cell cultures, is the absence of blood flow and infiltrating immune cells. Nevertheless, they provide a snapshot of the neurovascular unit up to the time of brain isolation if a lesion and/or treatment is applied *a priori*. Younger animals such as 2-day-old mice still grant the ability to inject a therapeutic agent *via* the temporal vein, which is still visible at this age, to study its protective value (Machado-Pereira et al., 2018). Subsequently, therapeutic agents and stimuli can be further administered over tissue to evaluate their impact on neurovascular and glial activity.

## Animal models of perinatal stroke

Experimental models are important to understand mechanisms of disease. Parameters like injury onset and duration, area of penumbra, reperfusion or therapeutic window are clearly defined, and symptoms can be easily identified and monitored. However, reproducing all the complex pathophysiological aspects of stroke in an otherwise healthy animal is challenging; typically, a stroke patient is elderly and has more than one health condition. One of the most frequently used adult models is induced by transient occlusion of the middle cerebral artery (MCA) with an intraluminal monofilament, blocking cerebral blood flow (usually 60 min) and causing reproducible infarcts in this territory (Carmichael, 2005; Sommer, 2017). A transient model allows the study of the significant effects of reperfusion. Regarding the perinatal period, the most common methodologies use unilateral ligation of the common carotid artery followed by hypoxia, or direct exposure to hypoxia alone. Table 1 briefly describes procedures using rodents, rabbits, pigs and lambs. Animal models employing pigs, lambs or rabbits, are costly in terms of maintenance, in the sense that they have longer gestation periods and smaller litter size, while offering higher genetic dissimilarity with humans, comparing with mice (Leong et al., 2015). Importantly, most models use O_2_ rates much higher (5–12%) than those believed to occur after an ischemic event. Only one group used a lower percentage (3.5–4%), albeit on rats of an age range comparable to a term and up to 2-years-old infant. In fact, normal brain tissue pO_2_ is 33.8 ± 2.6 mmHg, which corresponds to 4.4 ± 0.3% O_2_ in the microenvironment (Carreau et al., 2011). Three of the models used between 6 and 12% O_2_: a fraction of inspired oxygen (FiO_2_) of 0.06–0.12. Considering that atmospheric air is 21% O_2_ or the equivalent to a FiO_2_ of 0.21, these animals would be subjected to a third to a half O_2_ available. Are lower O_2_ rates fatal? Moreover, most models use 7-days-old or older animals, which offer a more reasonable size for surgery than younger pups. A week old rat, the most commonly used species, would represent a 2-months-old infant (well-beyond the 28th day post-birth) if considering peripheral organ systems (Sengupta, 2013; Titomanlio et al., 2015), and a term infant, if considering brain development (Titomanlio et al., 2015), raising further challenges on what age range to choose.

**Table 1:** Perinatal and neonatal animal models for ischemic injury, specifically rat, pig, mouse, rabbit, and lamb.

**RAT**		
P5-P17 Φ	Exposure to hypoxia (3.5–4% O_2_ in N_2_) until apnea or heart rate below 20% of baseline	Jensen, 1995
P10 Φ	Exposure to hypoxia (7, 5, 4% O_2_ in N_2_) for 8, 6, and 1 min, respectively	Dunn et al., 2017
P10 Φ	Left MCAO for 90 min by inserting a 6-0 nylon filament into the internal carotid artery; unilateral ligation of right CCA followed by hypoxia (8% O_2_ in N_2_) for 90 min	Ashwal et al., 2007
P7 ♀	MCAO for 180 min by inserting a 6-0 coated filament into the internal carotid artery	Fernández-López et al., 2013
P7 Φ	Unilateral ligation of right CCA followed by hypoxia (8% O_2_ in N_2_) for 60 min	Jantzie and Todd, 2010
P7 Φ	Unilateral ligation of CCA followed by hypoxia (8% O_2_ in N_2_) for 30, 60, 90, or 120 min	Silverstein and Johnston, 1984
P7 Φ	Unilateral ligation of left CCA followed by hypoxia (8% O_2_ in 92% N_2_) for 90 min	Bae et al., 2012
P7 Φ	Unilateral ligation of right CCA followed by hypoxia (8% O_2_ in N_2_) for 180 min	Jantzie et al., 2005
P7 Φ	Unilateral ligation of left CCA followed by hypoxia (8% O_2_ in N_2_) for 120 min	Lubics et al., 2005
P7 Φ	Unilateral ligation of CCA followed by hypoxia (8% O_2_ in N_2_) for 60–240 min	Vannucci and Vannucci, 2005
P4 ♂	Exposure to hypoxia (11% O_2_ in N_2_) for 360 min *per* day for 5 days	Schaeffer et al., 2013
P4 Φ	Exposure to hypoxia (11% O_2_ in N_2_) for 360 min *per* day for 5 days	Fendt et al., 2008
P2 Φ	Exposure to hypoxia (12% O_2_ in N_2_) for 14 days	Deruelle et al., 2006
P1 ♂	Exposure to hypoxia (12% FiO_2_) for 10 days	Del Duca et al., 2009
P1 Φ	Unilateral ligation of right CCA followed by hypoxia (8% O_2_ in N_2_) for 210 min	Girard et al., 2009
P1 Φ	Exposure to hypoxia (5% O_2_ in N_2_) for 60 or 75 min	Slotkin et al., 1995
Newborn ♂/♀	Unilateral ligation of right CCA followed by hypoxia (8% O_2_ in N_2_) for 120 min	Kartal et al., 2016
**PIG**		
P3-P7 Φ	Exposure to a gas mixture (10% FiO_2_) for 40 min, followed by 5 min of reoxygenation and 7 min of anoxia by clamping the endotracheal tube	Ni et al., 2011
P1-P4 ♂	Exposure to a gas mixture (FiO_2_ 6–8%) until hearth rate decreased to 60 beats/min (bradycardia) or mean arterial blood pressure decreased to 15 mmHg (severe hypotension)	Faa et al., 2012
P1-P3 Φ	Exposure to a gas mixture (12% O_2_ in N_2_) for 120 min to achieve a pO_2_ of 30–40 mmHg	Stevens et al., 2008
Newborn Φ	Exposure to a gas mixture (8% O_2_ in N_2_) until the mean arterial blood pressure decreased to 20 mmHg or base excess reached −20 mmol/L	Garberg et al., 2017
**MOUSE**		
P9 Φ	Unilateral ligation of left CCA followed by hypoxia (10% O_2_ in N_2_) for 60 min	Kichev et al., 2014
P7 ♂	Exposure to hypoxia (10% O_2_ in N_2_) for 360 min *per* day (3 sessions of 120 min separated by 45 min intervals) for 6 days	Kameda et al., 2013
**RABBIT**		
E22 Φ	Uterine ischemia for 40 min, *via* arterial embolectomy catheter inserted through the maternal left femoral artery into the descending aorta	Yu et al., 2011
**LAMB**		
E126-141 Φ	Intrauterine hypoxia by induced maternal hypotension *via* infusion of trimetaphan camsylate glucose solution into a polyethylene catheter placed in the maternal femoral vein, for 60–90 min	Gersony et al., 1976

*Rodent models usually resort to exposure to hypoxia alone (3.5–12% O_2_) or to unilateral ligation of the common carotid artery, followed by hypoxia (8% O_2_), in animals of varying ages. Pig models use exposure to a gas mixture ranging from 6 to 12% O_2_ while lamb and rabbit models induce intrauterine ischemia. ♀, female; ♂, male; Φ undisclosed sex; CCA, common carotid artery E, embryonic days; MCAO, middle cerebral artery occlusion; min, minutes; P, post-natal days*.

Age is very important, since the extent of ischemic injury is largely influenced by brain maturity (Sheldon et al., 1996; McQuillen et al., 2003; Webber et al., 2009). In preterm newborns, oligodendrocyte progenitor cells (OPC) are particularly more sensitive to ischemia (Back et al., 2002). Global ischemia, as in hypoxic-ischemia encephalopathy, disrupts OPC maturation, causing delayed or disrupted myelination, largely contributing to neuronal loss and periventricular white matter diffuse injury (periventricular leukomalacia; Back et al., 2007; Webber et al., 2009). Several experimental models have also been proposed to study this particular condition (Shen et al., 2010). For this reason, OPC constitute a potential target for the development of protective therapies focusing on the reduction of white matter loss in premature infants (Back et al., 2007). Subplate neurons, a transient neuronal population important for the formation of mature neuronal networks, are another vulnerable target. Additionally, interneuron migration to the neocortex is only completed at birth, in a process modulated by microglia activity (Leviton and Gressens, 2007; Xu et al., 2011). In term newborns, gray matter is focally affected, greatly impacting on motor function (Back et al., 2001; Fernández-López et al., 2014; Luhmann et al., 2016). The immature brain is also more susceptible to excitotoxicity and to free radicals considering the higher expression of receptors that signal for excitatory neurotransmitters and lower levels of anti-oxidant enzymes (Johnston, 2005; Lafemina et al., 2006). Overall, choosing the “right” age pertains to the fact that the perinatal period encompasses different stages of the circulatory and immune systems (Titomanlio et al., 2015; Lange et al., 2016). Accordingly, the therapeutic value of an agent directed at perinatal stroke would be better assessed using younger animals. Fundamentally, the brain vasculature during development is formed through two distinct processes: (i) vasculogenesis, in which angioblasts differentiate into endothelial cells forming the perineural vascular plexus, which in turn functions as a substrate for (ii) angiogenesis, the process of generating new vessels from pre-existing ones (Vasudevan and Bhide, 2008; Lee et al., 2009; Tam and Watts, 2010). These processes are consolidated by migrating mural cells, formation of an extracellular matrix and establishment of tight and adherens junctions that regulate permeability and transcellular transport (Lee et al., 2009; Tam and Watts, 2010). With increasing age, blood-brain barrier (BBB) functionality is less maintained after stroke since the expression of several of these proteins (e.g., occludin, claudins, zonula occludens proteins; Kratzer et al., 2014) that support the integrity of tight junctions is also changed. Another reason for a higher resistance of the BBB to ischemic injury in the early stages could be the maturation-dependent interplay between leukocytes and the endothelium and the less active pathophysiological role of the inflammatory process (Titomanlio et al., 2015). A restricted BBB opening may also account for limited neutrophil recruitment/infiltration. While regulatory T cells seem to play a neuroprotective role, microglia cells promote phagocytosis and tissue recovery, or white matter damage, depending on the adopted phenotype (Hagberg et al., 2015). In fact, microglia migrate to the brain even before blood vessel formation possibly impacting on the development of these structural elements (Rymo et al., 2011; Arnold and Betsholtz, 2013). Hence, in the perinatal period, the neuroinflammatory response has a preponderant role in stroke outcome (and to diffuse pattern of injury), relying more on the activation of microglia than on the extrinsic recruitment of inflammatory cells such as macrophages and neutrophils (Hagberg et al., 2015). A more mature brain offers a BBB more vulnerable to ischemic injury and to immune cell infiltration, and therefore, these cells assume a greater role and become associated to a focal pattern of injury. Consequently, several pro-/anti-inflammatory molecules and growth factors are released and have been studied as part of the impactful secretome unleashed by ischemia. Vascular endothelial growth factor (VEGF) is responsible for several processes upon ischemic stroke, including disruption of endothelial cell junctions and endothelial cell endocytosis, followed by increased BBB permeability, and consequently intracranial hemorrhage and intracranial hypertension (Angelo and Kurzrock, 2007; Lange et al., 2016; Suzuki et al., 2016). However, VEGF also promotes endothelial cell proliferation and migration, and enhances perfusion (reduced infarct volume and penumbra were associated to increased neuroprotection, including in neonatal stroke; Titomanlio et al., 2015; Lange et al., 2016; Suzuki et al., 2016). There are other cell types responsible for the development, regulation and maintenance of central nervous system angiogenesis and BBB integrity such as pericytes and astrocytes (Tam and Watts, 2010). These cells control the production and release of several factors that regulate the aforementioned processes (Lee et al., 2009; Tam and Watts, 2010; Arnold and Betsholtz, 2013). Therefore, diminished quantities of pericytes and astrocytes alongside blood vessels are associated to a higher susceptibility to ischemic injury (Fernández-López et al., 2014; Kratzer et al., 2014).

On a final note, rodent strains may display different levels of vulnerability to injury. For instance, CD1 mice are more vulnerable to damage induced by 30 min of hypoxia, than C57BL/6 and 129Sv mice, with the latter being the most resistant strain (Sheldon et al., 1998). Other murine strains (BDF, CFW, and BALB/C) display varying infarct volumes following 24 h of focal ischemia possibly because of differences in the anatomy of the posterior communicating arteries. BALB/C mice showed a more significant infarct volume and were proposed as the most suitable strain to conduct pharmacological studies in cerebral ischemia (Barone et al., 1993). In addition to differences in vascular anatomy, humans and other animals also display significantly different nutrient and oxygen metabolism, hemodynamics, and neural cell population density/activity (Dirnagl et al., 1999).

An interesting but poorly studied subject is the fact that perinatal stroke appears to be gender-dependent with male neonates and children being more commonly affected and with poorer outcomes (Turtzo and McCullough, 2010; Fernández-López et al., 2014). Turtzo and McCullough have extensively reviewed the role of gender and sex hormones in the perinatal, infant and adult periods. Although *in vitro* data from female pups suggest higher protection from OGD, and neuronal cells from males seem more susceptible to hypoxic injury (Heyer et al., 2005; Li et al., 2005), clinical studies and mechanisms of action remain inconclusive. Overall, ischemic cell death occurs *via* a caspase-independent pathway in males, while this process is caspase-dependent in females; ultimately, both pathways lead to mitochondrial dysfunction. The protection provided by female sex hormones, such as estrogen and progesterone, is a possible explanation. However, estrogen and progesterone administration to post-menopausal women were found to raise the risk of stroke (Turtzo and McCullough, 2010). To further investigate this issue in the perinatal period, it is possible to divide pups, even after birth, by a distinct physical trait: male mice have a visible pigment spot on the scrotum (Wolterink-Donselaar et al., 2009).

## Conclusions

*In vitro* models are useful for assessing the potential of a therapeutic agent and constitute an inescapable stepping stone for *in vivo* models. However, current animal models still hold key limitations regarding the level of hypoxia and extent of focal injury, age and costs associated to the selected animal species/strain, as well as their basic anatomy. Importantly and understandably, all fail to reproduce the exact mechanisms of injury that occur specifically in perinatal stroke. Hence, it is urgent to continue advancing newer (and multifactorial) experimental models to attain more efficient therapies to treat this complex vascular condition and long-term sequela.

## Author contributions

All authors listed have made a substantial, direct and intellectual contribution to the work, and approved it for publication.

### Conflict of interest statement

The authors declare that the research was conducted in the absence of any commercial or financial relationships that could be construed as a potential conflict of interest.
