# Supplementary material for: The Ischemic Immature Brain: Views on Current Experimental Models
Source: Front Cell Neurosci. 2018 Aug 29;12:277. doi: 10.3389/fncel.2018.00277 (PMC6123378; doi:10.3389/fncel.2018.00277)
Supplement: Supplementary file 1 [file Table_1.DOCX]

| **Table 1: Risk factors for perinatal stroke** | **References** |
| --- | --- |
| **Maternal** |  |
| Autoimmune disorders | ([1](#_ENREF_1)) |
| Coagulation disorders: protein C or S deficiency, factor V Leiden, prothrombin mutation | ([1](#_ENREF_1); [2](#_ENREF_2); [3](#_ENREF_3)) |
| Twin-to-twin transfusion syndrome | ([1](#_ENREF_1)) |
| Acquired antiphospholipid antibodies | ([1](#_ENREF_1); [2](#_ENREF_2); [3](#_ENREF_3)) |
| Cocaine abuse or other vasoconstrictor (e.g. amphetamines, codeine) | ([1](#_ENREF_1); [2](#_ENREF_2); [4](#_ENREF_4); [5](#_ENREF_5)) |
| Infection: central nervous system infection, systemic infection | ([1](#_ENREF_1); [5](#_ENREF_5); [6](#_ENREF_6)) |
| Pre-eclampsia/eclampsia | ([2](#_ENREF_2); [4](#_ENREF_4); [6](#_ENREF_6)) |
| Prolonged rupture of membranes | ([6](#_ENREF_6)) |
| Gestational diabetes | ([2](#_ENREF_2)) |
| Fever | ([2](#_ENREF_2); [6](#_ENREF_6); [7](#_ENREF_7)) |
| History of infertility | ([6](#_ENREF_6)) |
| **Fetal/Neonatal** |  |
| Cardiac disorders: congenital heart disease, patent ductus arteriosus, pulmonary valve atresia | ([1](#_ENREF_1); [2](#_ENREF_2); [3](#_ENREF_3); [4](#_ENREF_4); [8](#_ENREF_8)) |
| Blood and lipid disorders: polycythemia, prothrombin mutation, factor V Leiden, factor VIII, protein C or S deficiency, hyperhomocysteinemia, antithrombin deficiency, methyltetrahydrofolate reductase gene mutation, increased lipoprotein(a), disseminated intravascular coagulopathy | ([1](#_ENREF_1); [2](#_ENREF_2); [3](#_ENREF_3); [4](#_ENREF_4); [5](#_ENREF_5)) |
| Vascular malformation or defect | ([1](#_ENREF_1); [4](#_ENREF_4); [5](#_ENREF_5); [8](#_ENREF_8)) |
| Uterine growth restriction | ([4](#_ENREF_4)) |
| Hypoglycemia | ([2](#_ENREF_2); [6](#_ENREF_6)) |
| Infection: central nervous system infection, systemic infection | ([1](#_ENREF_1); [2](#_ENREF_2); [4](#_ENREF_4); [5](#_ENREF_5); [6](#_ENREF_6); [8](#_ENREF_8)) |
| Hyperthermia | ([7](#_ENREF_7)) |
| Nephrotic syndrome | ([5](#_ENREF_5)) |
| Apgar score <7 at 5 minutes | ([2](#_ENREF_2); [6](#_ENREF_6)) |
| **Placental** |  |
| Placental thrombosis | ([1](#_ENREF_1); [2](#_ENREF_2)) |
| Placental abruption | ([1](#_ENREF_1); [2](#_ENREF_2)) |
| Placental infection | ([1](#_ENREF_1); [2](#_ENREF_2); [6](#_ENREF_6)) |
| Feto-maternal hemorrhage | ([1](#_ENREF_1); [2](#_ENREF_2)) |
| **Other** |  |
| Trauma and catheterization | ([1](#_ENREF_1); [4](#_ENREF_4); [5](#_ENREF_5)) |
| Birth asphyxia | ([1](#_ENREF_1); [2](#_ENREF_2); [4](#_ENREF_4); [5](#_ENREF_5)) |
| Dehydration | ([1](#_ENREF_1); [4](#_ENREF_4)) |
| Extracorporeal membrane oxygenation | ([1](#_ENREF_1); [5](#_ENREF_5)) |

List of references

[1] R.S. Gunny, and D. Lin, Imaging of perinatal stroke. Magnetic resonance imaging clinics of North America 20 (2012) 1-33.

[2] D. Fernandez-Lopez, N. Natarajan, S. Ashwal, and Z.S. Vexler, Mechanisms of perinatal arterial ischemic stroke. Journal of cerebral blood flow and metabolism : official journal of the International Society of Cerebral Blood Flow and Metabolism 34 (2014) 921-32.

[3] M.H. Cnossen, C.H. van Ommen, and I.M. Appel, Etiology and treatment of perinatal stroke; a role for prothrombotic coagulation factors? Seminars in fetal & neonatal medicine 14 (2009) 311-7.

[4] V. Machado, S. Pimentel, F. Pinto, and J. Nona, Acidente vascular cerebral isquêmico perinatal: estudo retrospectivo de 5 anos em maternidade nível III. Einstein (16794508) 13 (2015).

[5] P. Govaert, L. Ramenghi, R. Taal, J. Dudink, and M. Lequin, Diagnosis of perinatal stroke II: mechanisms and clinical phenotypes. Acta paediatrica 98 (2009) 1720-6.

[6] H. Hagberg, C. Mallard, D.M. Ferriero, S.J. Vannucci, S.W. Levison, Z.S. Vexler, and P. Gressens, The role of inflammation in perinatal brain injury. Nat. Rev. Neurol. 11 (2015) 192-208.

[7] E. Kasdorf, and J.M. Perlman, Hyperthermia, inflammation, and perinatal brain injury. Pediatric neurology 49 (2013) 8-14.

[8] S.E. Buerki, D. Grandgirard, A.N. Datta, A. Hackenberg, F. Martin, T. Schmitt-Mechelke, S.L. Leib, M. Steinlin, and G. Swiss Neuropediatric Stroke Registry Study, Inflammatory markers in pediatric stroke: An attempt to better understanding the pathophysiology. Eur J Paediatr Neurol 20 (2016) 252-260.
